# Lithium as a Potential Senostatic Agent in Central Nervous System Aging and Bipolar Disorder

**DOI:** 10.3390/ph19091378

**Published:** 2026-08-31

**Authors:** Janusz K. Rybakowski, Cezary Mydlak, Nadzieja Klimek, Krzysztof Książek

**Affiliations:** 1 Department of Adult Psychiatry, Poznań University of Medical Sciences, Szpitalna 27/33, 60-572 Poznań, Poland; 2 Department of Pathophysiology of Ageing and Civilization Diseases, Poznań University of Medical Sciences, Święcickiego 4 Str., 60-781 Poznań, Poland; cezary.mydlak@ump.edu.pl; 3 Faculty of Medical Sciences, Prince Mieszko I Poznan Medical University of Applied Sciences, Bulgarska 55 Str., 60-320 Poznań, Poland; nadziejaklimek20@gmail.com (N.K.); k.ksiazek@pam.poznan.pl (K.K.)

**Keywords:** bipolar disorder, brain aging, lithium, senotherapy, neuroprotection

## Abstract

Lithium remains the gold-standard maintenance treatment for bipolar disorder (BD) and is distinguished by its unique anti-suicidal and neuroprotective properties. Beyond its established psychiatric efficacy, growing evidence suggests that lithium may modulate fundamental mechanisms of biological aging. In parallel, cellular senescence has emerged as a central process linking oxidative stress, chronic inflammation, mitochondrial dysfunction, and impaired cellular resilience with neurodegeneration and psychiatric disease. Notably, BD is increasingly associated with features of accelerated aging, including telomere shortening, increased inflammatory burden, and structural brain changes consistent with premature brain aging. In this review, we discuss current evidence supporting lithium as a potential senostatic agent in the central nervous system. Experimental studies indicate that lithium attenuates several hallmarks of cellular senescence and promotes cellular resilience under conditions of oxidative, inflammatory, and genotoxic stress. These effects appear to involve coordinated modulation of neuroinflammatory signaling, mitochondrial function, oxidative stress responses, genomic stability, and neurotrophic pathways. Clinical findings further suggest that chronic lithium treatment may be associated with preserved telomere length and attenuated biological brain aging in BD. Collectively, the available data support a model in which lithium acts not only as a mood stabilizer but also as a broader regulator of aging-associated processes relevant to neuropsychiatric and neurodegenerative disorders. Although the senomodulatory effects of lithium appear context- and cell-type-dependent, its established clinical use and pleiotropic biological actions make it a promising candidate for translational senotherapeutic research.

## 1. Senescence and Brain Aging

### 1.1. Overview of Cellular Senescence

Cellular senescence is a stress response characterized by permanent cell cycle arrest and extensive phenotypic and molecular alterations. It is induced by factors such as DNA damage, telomere shortening, oxidative stress, and oncogenic signaling. In addition to replicative senescence, stress-induced premature senescence (SIPS) can arise independently of telomere length, typically through persistent activation of the DNA damage response (DDR).

At the molecular level, senescence is primarily governed by the p53/p21 and p16/Rb pathways, which enforce stable cell-cycle arrest. Sustained DDR signaling via ataxia-telangiectasia mutated (ATM) and ATM and Rad3-related (ATR) stabilizes p53 and induces p21, while p16 inhibits CDK4/6, maintaining retinoblastoma protein (Rb) activity and repressing E2F-dependent transcription. These processes are accompanied by chromatin remodeling, including heterochromatin formation and epigenetic changes, as well as alterations in the nuclear envelope, such as loss of lamin B1. Mitochondrial dysfunction, increased reactive oxygen species (ROS), impaired mitophagy, and activation of pro-survival pathways further stabilize the senescent phenotype [1,2].

Given its heterogeneity, senescence cannot be identified by a single marker and requires integrated assessment. Common features include SA-β-Gal activity, increased expression of p16 and p21, and persistent DNA damage foci (γ-H2A.X, 53BP1). Additional supportive indicators comprise lamin B1 loss, lipofuscin accumulation, elevated lysosomal content, HMGB1 relocalization, and selected surface markers (e.g., DPP4, uPAR) [3,4].

A defining functional feature of senescent cells is the senescence-associated secretory phenotype (SASP), which includes cytokines (e.g., IL-6, IL-8), chemokines, growth factors, matrix-remodeling enzymes, and extracellular vesicles. SASP is regulated by pathways such as NF-κB, C/EBPβ, mTOR, cGAS-STING, and JAK/STAT. While transient SASP can support tissue repair and immune-mediated clearance of damaged cells, chronic SASP activation contributes to sustained inflammation, tissue dysfunction, and the propagation of senescence in neighboring cells [5]. With age, senescent cells accumulate due to cumulative damage and reduced immune surveillance, impairing tissue function and contributing to cardiovascular disease, type 2 diabetes, osteoarthritis, pulmonary fibrosis, chronic kidney disease, and neurodegeneration [6].

### 1.2. Cellular Senescence in Neurons and Glial Cells

Cellular senescence in the central nervous system (CNS) is a heterogeneous, cell-type-specific process that extends beyond classical proliferative arrest and reshapes tissue homeostasis. Brain aging involves region-specific vulnerability, synaptic remodeling, reduced dendritic complexity, and impaired proteostatic and metabolic resilience, accompanied by the accumulation of senescent cells that promote neuroinflammation and cognitive decline. CNS senescence reflects both post-mitotic stress adaptation in neurons and canonical senescence in proliferative glial cells [7,8].

Neurons develop a senescence-like phenotype in response to chronic genotoxic, oxidative, and metabolic stress. Although post-mitotic, aged neurons exhibit persistent DNA damage signaling and increased p21 expression [9,10], these changes are independent of cell-cycle re-entry. This state is associated with mitochondrial dysfunction, impaired autophagy, lipofuscin accumulation, elevated ROS, defective mitophagy, and altered NAD^+^ metabolism. Neurons also display chromatin remodeling, lamin B1 loss, cytoplasmic chromatin fragments, and activation of cGAS–STING signaling. Functionally, they adopt a SASP-like secretory phenotype, producing IL-6, IL-8, CCL2, and matrix remodeling enzymes, thereby contributing to local inflammation [8,9]. However, markers such as SA-β-Gal and lipofuscin should be interpreted cautiously in post-mitotic cells. Overall, neuronal senescence-like states represent maladaptive stress responses rather than true cell-cycle arrest.

In contrast, glial cells—including astrocytes and microglia—undergo canonical senescence due to their retained proliferative capacity. This is characterized by increased expression of p16INK4a and p21, persistent DDR, epigenetic remodeling, and robust SASP. Senescent astrocytes exhibit impaired glutamate uptake, disrupted metabolic support, altered lipid homeostasis, and reduced synaptogenic capacity [11,12,13]. Senescent microglia exhibit dystrophic morphology, impaired phagocytosis, and heightened inflammatory responses, including increased secretion of IL-1β, IL-6, TNF-α, and complement components. These phenotypes are accelerated by pathological stimuli, including amyloid-β, tau, α-synuclein, irradiation, oxidative stress, and toxins [12,13]. Mechanistically, NF-κB, mTOR, and cGAS–STING integrate stress signals into sustained SASP output [14].

Oligodendrocyte progenitor cells (OPCs) are also vulnerable to stress-induced senescence. Senescent OPCs adopt a pro-inflammatory phenotype and impair neuronal excitability via CCL3/CCL5–CCR5 signaling [15]. Given the importance of white matter integrity, impaired remyelination may contribute to both neurodegenerative progression and psychiatric symptoms [16]. In multiple sclerosis, senescent-like microglia and progenitor cells in demyelinating lesions are associated with reduced remyelination and functional decline [17,18]. Senescent glia also exert non-cell-autonomous toxicity, inducing synaptic dysfunction and neuronal death [12].

Accumulation of senescent glia in vivo correlates with neurodegeneration, including synaptic loss, tau pathology, and cognitive decline [19,20]. In Alzheimer’s disease (AD) and Parkinson’s disease (PD), increased p16INK4a-positive glial cells, persistent DNA damage signaling, and SASP are observed in human and animal studies [11,12,21]. Senescent microglia and astrocytes show heightened pro-inflammatory activity and impaired homeostatic functions, including reduced phagocytosis and metabolic support [12,13]. Additionally, mitochondrial dysfunction and NLRP3 inflammasome activation amplify IL-1β signaling, disrupting synaptic plasticity and memory [22,23]. Reducing senescent cell burden alleviates neuroinflammation and improves neuronal function in experimental models [24]. At the systems level, persistent SASP disrupts blood–brain barrier integrity and stem cell niches and promotes paracrine senescence [12].

Overall, CNS senescence integrates DNA damage, mitochondrial dysfunction, epigenetic instability, and inflammation across neuronal and glial populations. Neurons exhibit senescence-like stress adaptation, whereas glia undergo canonical senescence. Their accumulation links brain aging with neurodegenerative disease through chronic inflammation and impaired regeneration.

### 1.3. Senescence as a Systems-Level Amplifier of Neuroinflammation

Cellular senescence remodels the CNS neuroimmune architecture, shifting transient inflammation into chronic, self-sustaining circuits. In the aging brain, the accumulation of senescent neurons, glia, progenitors, and vascular cells forms a persistent inflammatory niche characterized by sustained DAMP signaling, impaired immune resolution, and extracellular matrix remodeling [8,22,23]. This is driven by persistent activation of the DNA damage response and cytoplasmic chromatin accumulation, resulting from nuclear envelope instability and lamin B1 loss, thereby activating the cGAS–STING pathway and inducing type I interferons and an NF-κB-dependent SASP [25,26]. This creates a feed-forward loop in which inflammatory signaling propagates paracrine senescence and amplifies tissue-level dysfunction. Mitochondrial dysfunction further contributes to this axis by releasing mitochondrial DNA and increasing reactive oxygen species, both of which act as endogenous DAMPs [22].

Inflammasome activation represents an additional link between senescence and neuroinflammation. Mitochondrial stress and cytosolic DNA promote NLRP3 activation in microglia, leading to caspase-1-dependent maturation of IL-1β and IL-18 [22,23,27]. Persistent inflammasome signaling drives chronic innate immune activation. Senescence also alters microglial phenotypes by disrupting receptor signaling, including TREM2-dependent pathways that affect metabolism and phagocytosis [28]. In SASP-rich environments, microglia show reduced debris clearance, heightened inflammatory responses, and impaired synaptic surveillance [12,13,17]. Complement components (C1q, C3) further contribute to aberrant synaptic elimination and progressive synaptic loss [29].

At the neurovascular interface, endothelial and pericyte senescence promotes blood–brain barrier (BBB) dysfunction, increasing permeability to peripheral immune mediators and amplifying CNS inflammation [30]. Systemic inflammaging, marked by elevated IL-6 and TNF-α, interacts bidirectionally with CNS senescence, thereby sustaining immune activation [22,23]. Senescence also impairs the resolution of neuroinflammation. Resistance to apoptosis and reduced immune clearance prolong inflammatory signaling and disrupt regeneration [8,23]. Hippocampal neurogenesis declines in pro-inflammatory environments, and oligodendrocyte progenitor differentiation is impaired in demyelinating conditions [15,18]. In neurodegenerative diseases, senescence-driven inflammatory circuits synergize with proteinopathies to accelerate neuronal vulnerability and network dysfunction [8,31].

### 1.4. Cellular Senescence Linking Neurodegeneration and Psychiatric Disorders

Emerging evidence indicates that cellular senescence is a convergent process linking chronic neuroinflammation with circuit-level dysfunction in neurodegenerative and neuropsychiatric disorders [7,8,31]. Rather than an isolated phenotype, senescence disrupts neuronal–glial communication, synaptic stability, and immune–brain crosstalk, influencing cognitive and behavioral outcomes [8,9]. The role of cellular senescence is most evident in age-related neurodegeneration; however, accumulating evidence suggests that similar mechanisms may also contribute to psychiatric disorders, including schizophrenia, bipolar disorder (BD), and major depression [22,23,32]. This association is partly mediated by immune senescence, which links these conditions through chronic inflammation and dysregulated immune function [23,33].

For example, older adults with major depressive disorder exhibit elevated levels of SASP factors compared to cognitively normal, never-depressed controls [34]. Moreover, an exacerbated SASP has been associated with poorer cognitive performance [35], reduced response to antidepressant treatment [36], and worse physical health outcomes [37]. Major depressive disorder is also linked to elevated inflammatory markers, microglial activation, and NLRP3 signaling [38,39]. Cytokines such as IL-1β and TNF-α impair synaptic plasticity, reduce brain-derived neurotrophic factor (BDNF), and disrupt neurogenesis [38,40]. Although direct evidence of CNS senescence is limited, peripheral immune senescence—such as T-cell exhaustion—has been reported and may affect the brain via cytokine signaling and metabolic pathways [33,41].

Senescence may contribute to psychiatric disease by amplifying genomic instability, mitochondrial dysfunction, inflammasome activation, complement signaling, and immune aging into a persistent inflammatory state [8,22,31]. In neurodegeneration, this accelerates neuronal loss [12,21], while in psychiatric disorders, it may influence vulnerability and disease course through effects on neuroplasticity [23,33].

### 1.5. Senescence as a Key Mechanism of Accelerated Aging in Psychiatric Disorders

Major psychiatric disorders, such as schizophrenia, BD, and major depression, are associated with reduced life expectancy by 10–20 years [42]. This is due to increased somatic morbidity and premature mortality, including suicide. Accelerated aging may also partly explain these phenomena; all these illnesses exhibit features of accelerated aging. For example, according to a recent review, schizophrenia, BD, and major depression present accelerated epigenetic aging based on predefined DNA methylation patterns [43].

Of these three conditions, the data on accelerated aging in BD will be presented in more detail, as lithium, the drug discussed here, is primarily therapeutic in this condition. Apart from the epigenetic alterations mentioned above, the mechanisms in BD may include, among others, telomere attrition, mitochondrial dysfunction, and inflammation. Oxidative stress is considered a major driver of telomere shortening [44], a process frequently associated with BD and a potential marker of accelerated biological aging [45]. Research on telomere length in BD has shown that BD patients tend to have significantly shorter telomeres than healthy controls [46,47,48]. Interestingly, telomere shortening has also been observed in unaffected siblings of BD patients, suggesting that impaired telomere maintenance may either represent a vulnerability factor for BD or reflect the strong heritability of telomere dynamics [49]. Importantly, telomere shortening in BD occurs alongside other markers of accelerated biological and cellular aging. Patients with BD were also found to exhibit multiple hallmarks of premature aging, including increased brain-predicted age difference (brain-PAD), elevated SASP indices, and altered mitochondrial DNA copy number [44,48,50,51,52]. Notably, elevated SASP levels in older adults with BD were associated with greater depressive symptom severity and higher somatic disease burden, further supporting the concept of enhanced senescence-associated inflammatory activity in BD.

### 1.6. Senotherapeutic Strategies: From Systemic Targeting to CNS Applications

Cellular senescence, as a modifiable driver of aging, has led to “senotherapy,” which targets senescent cells by eliminating them (senolytics) or by suppressing harmful SASP effects (senomorphics), thereby reducing tissue dysfunction without inducing cell death [7]. Senolytics target senescent cells’ reliance on pro-survival pathways (e.g., BCL-2, PI3K/AKT). In animal models, their clearance reduces fibrosis, improves metabolism and function, and extends healthspan. Agents such as navitoclax, dasatinib plus quercetin, and FOXO4–p53 peptides show efficacy, supporting the role of senescent cells as active drivers of dysfunction [53,54,55]. In parallel, senomorphic strategies reduce pro-inflammatory SASP components without removing cells [56]. Targeting mTOR, NF-κB, JAK/STAT, or inflammasome pathways lowers inflammation and improves tissue function in aging models [57]. These approaches may offer advantages in contexts where complete ablation of senescent cells is impractical or potentially deleterious.

Translating senotherapeutic strategies to the CNS is challenging due to differences between senescence-like states in neurons and proliferative glia, as well as BBB limitations and the need for cell-specific targeting [8,9,10]. Nevertheless, reducing senescent glial burden or SASP-driven neuroinflammation has been shown to restore synaptic function, enhance remyelination, and improve cognition in preclinical models [12].

Within the CNS, senescence interacts with mitochondrial dysfunction, DNA damage, cGAS–STING, complement signaling, and NLRP3 activation [58]. These pathways offer targets for modulating neuroinflammation without cell loss, highlighting agents that affect autophagy, GSK-3, mitochondria, and inflammatory transcription [15,23,31]. This convergence raises the question of whether neuropsychiatric drugs can modulate senescence pathways.

Lithium, used for mood stabilization and with neuroprotective properties, targets these mechanisms and may influence senescence-driven neuroinflammation in the aging brain [59]. The suggestion of lithium’s anti-aging effect was first put forward by Salarda et al. [60]. In this narrative review, we critically examine the available experimental, translational, and clinical evidence regarding lithium as a potential senostatic agent, with particular emphasis on the central nervous system and bipolar disorder. Particular attention is given to evidence on cellular senescence biomarkers, oxidative and genomic stress, inflammatory signaling, glial responses, and markers of biological aging that may be modulated by lithium.

For the purposes of this review, evidence for a potential senostatic effect was considered strongest when lithium was evaluated in an established model of cellular senescence and was shown to attenuate multiple complementary features of the senescent phenotype, including senescence-associated β-galactosidase activity, expression of p16 and/or p21, persistent DNA damage signaling, SASP-related changes, or senescence-associated functional impairment, without evidence of selective elimination of senescent cells. Studies demonstrating modulation of oxidative stress, DNA repair, telomere maintenance, mitochondrial function, or inflammatory signaling without direct assessment of cellular senescence were considered supportive or indirect evidence. Clinical measures such as telomere length, TERT expression, or brain-PAD were interpreted as translational indicators of biological aging rather than direct evidence of cellular senescence.

### 1.7. Objectives of the Review

The primary objective of this review is to critically evaluate the evidence supporting lithium as a potential senostatic agent, with particular emphasis on the central nervous system and bipolar disorder. Specifically, we aim to: (i) summarize experimental evidence on the effects of lithium on established markers and phenotypes of cellular senescence; (ii) examine molecular mechanisms that may contribute to senescence modulation, including oxidative stress, DNA damage and repair, mitochondrial homeostasis, inflammatory signaling, SASP regulation, and neurotrophic pathways; (iii) assess translational and clinical findings related to biological aging, including telomere dynamics, TERT expression, and brain-predicted age difference; (iv) distinguish direct evidence of senostatic activity from indirect or supportive mechanistic evidence; and (v) identify current limitations, inconsistencies, and priorities for future research.

### 1.8. Review Methodology and Criteria for Senostatic Activity

This structured narrative review was based primarily on searches of PubMed/MEDLINE covering publications relevant to lithium, cellular senescence, brain aging, and bipolar disorder. The search combined the term “lithium” with terms related to cellular senescence and biological aging, including “cellular senescence,” “senostatic,” “senomorphic,” “senotherapy,” “brain aging,” “bipolar disorder,” “neuroinflammation,” “senescence-associated secretory phenotype” or “SASP,” “oxidative stress,” “DNA damage,” “DNA repair,” “telomere,” “telomerase” or “TERT,” “mitochondrial dysfunction,” “microglia,” “astrocytes,” “GSK-3,” “cGAS-STING,” “NLRP3,” and “brain-predicted age difference” or “brain-PAD.” Reference lists of relevant reviews and primary studies were also manually screened to identify additional publications directly related to the review’s scope.

Because direct evidence specifically examining lithium as a senostatic agent remains limited, the review incorporated experimental, translational, and clinical evidence at different levels of biological organization. Studies directly assessing cellular senescence were given the greatest weight when they evaluated lithium in established or experimentally induced senescence models and measured recognized features of the senescent phenotype. These included senescence-associated β-galactosidase (SA-β-Gal) activity, p16 and p21 expression, persistent DNA damage signaling, senescence-associated heterochromatin changes, SASP-related alterations, or senescence-associated functional impairment.

For the purposes of this review, evidence for a potential senostatic effect was considered strongest when lithium attenuated one or more established features of the senescent phenotype without evidence that the observed effect resulted from the selective elimination of senescent cells. Particular weight was given to studies assessing multiple complementary senescence-associated endpoints. In contrast, studies demonstrating modulation of oxidative stress, mitochondrial function, DNA repair, inflammatory signaling, autophagy, or other pathways mechanistically associated with cellular senescence, but without direct assessment of senescence markers, were interpreted as supportive or indirect evidence rather than proof of senostatic activity.

Clinical and translational studies were included when they investigated biological aging-related outcomes potentially relevant to senescence, including telomere length, telomerase reverse transcriptase (TERT) expression, inflammatory or SASP-related biomarkers, and brain-predicted age difference (brain-PAD). These outcomes were interpreted as indicators of biological aging or senescence-associated processes rather than as direct evidence of cellular senostasis. Particular caution was applied when extrapolating findings from peripheral biomarkers to cellular processes within the central nervous system.

Preclinical studies using neuronal, astrocytic, microglial, endothelial, or other cellular models were included when they provided direct evidence of lithium-related modulation of senescence or mechanistic evidence relevant to senescence-associated pathways. Animal studies were used to assess effects on neuroinflammation, oxidative stress, genomic stability, mitochondrial function, and neurodegenerative phenotypes. Clinical studies in bipolar disorder, mild cognitive impairment, Alzheimer’s disease, and other relevant populations were considered primarily as translational evidence regarding biological aging and neuroprotective effects. Evidence derived from models outside the central nervous system was included only when it addressed a mechanism directly relevant to the interpretation of lithium’s effects on cellular senescence and was explicitly treated as indirect or context-specific evidence.

Potentially relevant publications were evaluated based on their alignment with the predefined scope of the review, the biological model, lithium exposure, senescence-related outcomes, and mechanistic or translational significance. Because this was a narrative rather than a systematic review, no formal risk-of-bias assessment, PRISMA-based study selection procedure, or quantitative synthesis of evidence was performed.

When interpreting the available evidence, studies were therefore considered according to their evidentiary proximity to cellular senostasis. Direct evidence comprised studies in which lithium was administered in an established senescence model and recognized senescence-associated phenotypes were measured. Supportive mechanistic evidence comprised studies examining pathways strongly linked to senescence, such as oxidative and genomic stress, mitochondrial dysfunction, inflammatory signaling, or cellular resilience, without direct assessment of senescence. Clinical and neuroimaging findings were regarded as translational evidence of biological aging and were not interpreted as demonstrating cellular senostasis per se.

The review is limited by the relatively small number of studies directly evaluating lithium in established cellular senescence models; substantial heterogeneity in experimental systems, lithium concentrations, and exposure durations; and the diversity of senescence-associated endpoints. Additional limitations arise from the inclusion of mechanistic and translational evidence that is indirectly related to cellular senescence, as well as from the restriction to English-language publications and the primary reliance on a single bibliographic database. Accordingly, the available evidence does not permit lithium to be definitively classified as a senostatic agent, and mechanistic or clinical findings were interpreted according to the strength and directness of their relationship to cellular senescence.

## 2. Lithium in Psychiatry

### 2.1. From Historical Background to Current Clinical Applications

The psychiatric use of lithium dates back to the late 19th century, when Carl Lange reported its beneficial effects in periodic depression, based on the concept of “uric acid diathesis” [61]. Earlier, lithium salts were introduced for the treatment of gout. A major breakthrough came in 1949 with John Cade, who demonstrated the efficacy of lithium carbonate in the treatment of mania [62]. This finding marked the beginning of modern lithium therapy and stimulated further research into its role in mood disorders.

In the following decades, lithium’s therapeutic profile was progressively established. Its prophylactic effect in mood disorders was confirmed, leading to the recognition of long-term lithium treatment as a cornerstone in preventing both manic and depressive recurrences [63,64]. Its antidepressant properties were also demonstrated, and in later years, lithium became widely used as an augmentation strategy in treatment-resistant depression. At the same time, a distinct subgroup of patients, termed “excellent lithium responders” (ERs), was identified. These individuals achieve complete remission under lithium monotherapy and are typically characterized by a classical episodic course of illness and a family history of bipolar disorder [65,66].

Currently, the primary indication for lithium is the prevention of manic and depressive recurrences in mood disorders, particularly BD. This recommendation has been consistently confirmed in 21st-century meta-analyses demonstrating lithium’s superiority over placebo, especially in preventing manic episodes [67,68]. Lithium is also effective in the acute treatment of mania, with efficacy comparable to other mood stabilizers, although in more severe cases, it is often combined with antipsychotic drugs to achieve faster symptom control [69]. In depressive episodes, lithium monotherapy is not typically considered a first-line option; however, it plays a significant role as an adjunctive treatment, enhancing the efficacy of antidepressants in treatment-resistant depression and, in some cases, producing rapid clinical improvement [70,71].

Importantly, lithium has a well-documented anti-suicidal effect and is associated with reduced suicide risk and overall mortality in patients with mood disorders [72]. Beyond its psychiatric applications, lithium has also demonstrated antiviral activity, including effects against herpesviruses and potentially other viral infections [73,74]. In recent years, increasing attention has been given to its neuroprotective properties, with evidence suggesting a reduced risk of dementia and possible therapeutic effects in neurodegenerative disorders such as Alzheimer’s disease [74].

### 2.2. The Effect of Lithium on Morbidity, Mortality, and Brain Aging in Bipolar Disorder

Long-term lithium treatment in BD is associated with reduced morbidity and mortality. It has protective effects on the skeletal system, increasing bone mineral density and lowering the risk of osteoporosis, and on the cardiovascular system, including favorable effects on cardiac structure and reduced stroke risk [75,76,77,78]. Beyond this, lithium use has been linked to a lower risk of asthma and certain cancers, without increasing the risk of diabetes [79,80,81].

Large population studies confirm that patients with BD have markedly increased mortality, particularly due to suicide. Among mood stabilizers, lithium treatment is consistently associated with the greatest reduction in both suicide risk and overall mortality. These findings have been supported by a large nationwide cohort analysis in Taiwan, which demonstrated significantly lower mortality rates in lithium-treated patients compared with those not receiving lithium [82]. The most recent analysis of 15,384 health records of individuals with BD in Catalonia, Spain, found that lithium exposure was associated with a significantly lower mortality risk (RR = 0.69) compared with no exposure [83].

Recent research has also shown that chronic lithium treatment was associated with modulatory effects on aging biomarkers, particularly those related to telomeres [84], and with a reduction in brain-PAD [52].

### 2.3. Lithium’s Mechanisms of Mood Stabilization

Current evidence indicates that lithium exerts pleiotropic effects at the cellular and molecular levels. Its key mechanisms include modulation of electrolyte balance and membrane transport, regulation of second messenger systems—particularly the phosphatidylinositol (PI) pathway—and inhibition of glycogen synthase kinase-3β (GSK-3β), a key regulatory kinase.

Lithium also influences neurotransmission and exhibits antiviral properties. Recent molecular and in vitro studies further link lithium’s actions to major pathogenic processes implicated in bipolar disorder, including low-grade inflammation, mitochondrial dysfunction, circadian rhythm disturbances, gut–brain axis alterations, and purinergic signaling. Among these mechanisms, modulation of PI signaling and GSK-3β inhibition represent prominent and well-characterized actions of lithium, but they operate within a broader network of molecular effects [85].

### 2.4. Neuroprotection and Anti-Dementia Effects of Lithium

The first evidence of lithium-induced increases in brain grey matter emerged in 2000 [86]. Subsequent studies have shown that both short- and long-term lithium treatment can increase the volume of key regions, including the prefrontal cortex, anterior cingulate cortex, and hippocampus [87]. These findings support a neuroprotective role of lithium, further reinforced by population studies linking its use to reduced risk and severity of dementia [88]. In patients with BD, long-term treatment with lithium was associated with a reduced rate of dementia, in contrast to such treatment with anticonvulsants, antidepressants, and antipsychotics [89].

Clinical studies indicate that lithium may have beneficial effects in amnestic mild cognitive impairment (aMCI) and AD. In patients with aMCI, lithium treatment has been associated with reduced levels of hyperphosphorylated tau protein, stabilization of cognitive and functional performance, and a potential decrease in the rate of conversion to AD. In individuals with AD, lithium has been linked to preservation of global cognitive function and slower cognitive decline compared to untreated groups. Importantly, long-term follow-up data suggest that these benefits may persist, with improved cognitive outcomes, including better verbal fluency, observed in lithium-treated patients [90,91,92,93].

Further meta-analytic evidence in MCI and AD provides a more nuanced picture. Some studies report a slowing of cognitive decline and modest cognitive benefits associated with lithium, potentially influenced by treatment duration [94,95]. Analyses in larger patient cohorts with AD have also suggested favorable efficacy and tolerability compared with certain disease-modifying therapies [96]. However, more recent evidence indicates that, despite a satisfactory safety profile, lithium does not consistently translate into measurable cognitive or functional improvement in AD dementia [97].

**Figure 1:**
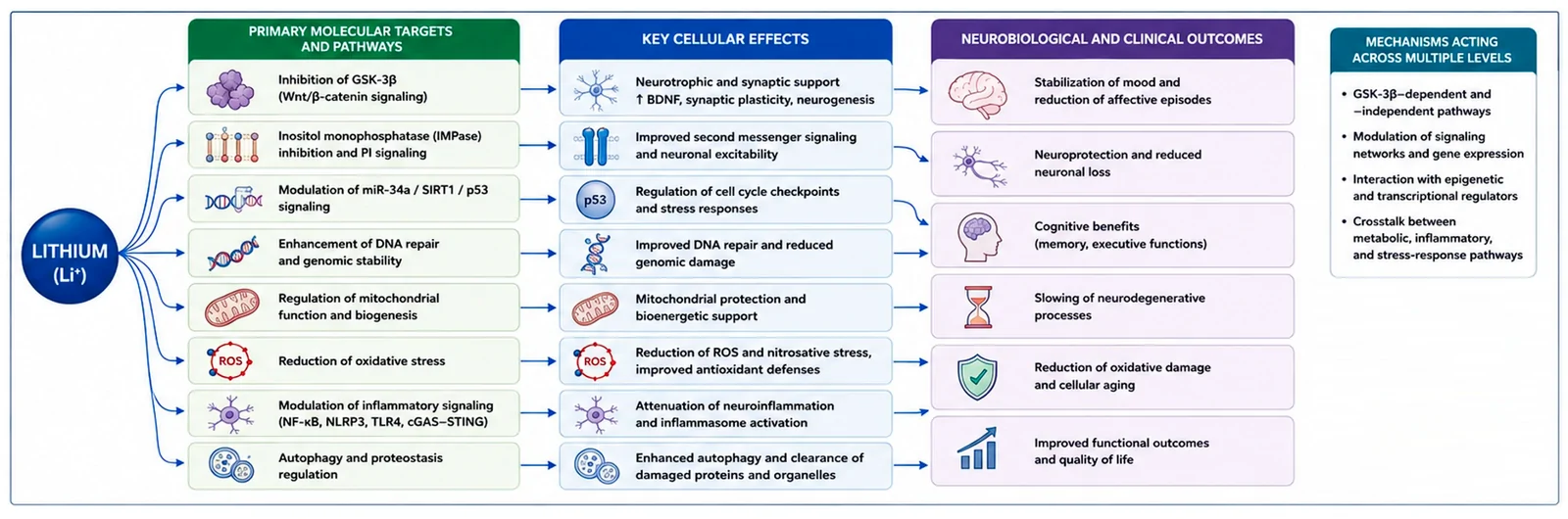
Multifaceted mechanisms of lithium action in bipolar disorder and neurodegeneration. Lithium exerts pleiotropic effects through multiple molecular and cellular pathways, including GSK-3β/Wnt–β-catenin signaling, phosphatidylinositol/inositol monophosphatase signaling, miR-34a/SIRT1/p53 regulation, DNA repair, mitochondrial function, oxidative stress modulation, inflammatory signaling, and autophagy/proteostasis. These interconnected mechanisms may contribute to neurotrophic support, improved cellular resilience, attenuation of neuroinflammation, and neuroprotective and cognitive effects. Solid arrows indicate direct or well-supported effects, whereas dashed arrows indicate indirect or modulatory relationships.

Emerging evidence also points to a relationship between lithium availability and dementia risk. Reduced endogenous lithium levels have been observed in the prefrontal cortex of individuals with cognitive impairment, while epidemiological studies show an inverse association between dementia prevalence and lithium concentration in drinking water [98,99,100]. These findings support a mechanistic explanation for lithium’s potential neuroprotective effects (Figure 1) and are consistent with a multifactorial model of lithium-induced neuroprotection. Proposed mechanisms include modulation of GSK-3β signaling, the phosphatidylinositol/inositol monophosphatase pathway, autophagy, mitochondrial function and biogenesis, intracellular and mitochondrial Ca^2+^ homeostasis, inflammatory signaling, and oxidative stress. Through these interconnected pathways, lithium may reduce tau phosphorylation and amyloid-related pathology, improve cellular bioenergetics and stress resilience, and support neuronal survival [73,101,102,103].

## 3. Evidence for Lithium as a Potential Senotherapeutic Agent

### 3.1. Effect on Senescence Biomarkers

There is mounting evidence that microdoses of lithium have beneficial effects on several symptoms of brain aging in patients with Alzheimer’s disease (AD) and in AD animal models. These benefits include improvements in memory maintenance, decreased senile plaque density, and reduced neuronal cell loss [91,104]. Experiments on human iPSC-derived astrocytes in vitro revealed that lithium reduced expression of p16 and p21 as well as reducing SA-β-Gal expression in astrocytes subjected to amyloid β-induced senescence [105]. Interestingly, these effects appeared independent of GSK-3β inhibition, as no significant changes in Ser9-GSK-3β phosphorylation were observed following lithium treatment. This finding suggests that the anti-senescent activity of lithium may be dose-dependent and mediated through distinct molecular pathways. Previous studies showed that high concentrations of lithium chloride (20 mM) reduced GSK-3-dependent nuclear accumulation of p53 and p21 in WI-38 fibroblasts [106], whereas findings at a much lower concentration (2.5 μM) appear to involve alternative cytoprotective mechanisms that regulate cellular senescence independently of classical GSK-3β signaling.

Additional evidence for lithium’s anti-senescent properties, including potentially non-canonical mechanisms of action beyond direct GSK-3β inhibition, comes from neuronal models of SIPS. In neuronal cells exposed to hydrogen peroxide-induced oxidative stress, lithium treatment reduced several hallmarks of senescence, including SA-β-Gal activity, lipofuscin content (according to Sudan Black B staining), and the formation of senescence-associated heterochromatin foci (SAHF). Lithium also attenuated cell-cycle arrest and modulated the expression of key senescence-related proteins, including p53, p21, p16INK4a, and SIRT1. Importantly, these effects were accompanied by alterations in the senescence-associated miR-34a/Sirt1/p53 signaling axis, suggesting that lithium-mediated suppression of neuronal senescence may involve epigenetic and post-transcriptional regulatory mechanisms [107]. The potential relevance of modulating miR-34a in this context is further supported by studies identifying it as a central pro-senescent and pro-inflammatory regulator in vascular aging. In vascular smooth muscle cells (VSMCs), increased miR-34a expression was associated with enhanced SASP activation, elevated IL-6 secretion, and promotion of osteoblastic differentiation and vascular calcification. Moreover, conditioned medium derived from miR-34a-overexpressing cells accelerated both senescence and calcification in neighboring cells, indicating that miR-34a may contribute to paracrine propagation of senescence-associated inflammatory signaling [108].

Counteraction of oxidative stress-related cellular damage by lithium may also involve stimulation of DNA repair mechanisms, particularly by enhancing non-homologous end-joining (NHEJ) pathways through upregulation of DNA ligase IV. In ischemic retinal neurocytes, lithium pretreatment reduced the number of γ-H2AX foci, indicating attenuation of DNA double-strand breaks, while simultaneously increasing ligase IV expression. Mechanistically, this effect involved cooperative transcriptional regulation by nuclear respiratory factor 1 (Nrf-1) and phosphorylated CREB1 (P-CREB1), both of which bind to the ligase IV promoter and enhance its transcription under ischemic conditions [109]. Consistent with these observations, lithium also accelerated repair of irradiation-induced DNA double-strand breaks in hippocampal neurons by enhancing DNA-dependent protein kinase (DNA-PK)-mediated NHEJ activity. Lithium treatment increased DNA-PK threonine 2609 foci formation and enhanced DNA end-joining activity, coinciding with reduced γ-H2AX accumulation and decreased neuronal apoptosis. Importantly, these neuroprotective effects were markedly attenuated following genetic or pharmacological inhibition of DNA-PK, further supporting a central role of NHEJ activation in lithium-mediated genomic protection [110]. In line with CREB-dependent signaling, lithium was also shown to enhance neuronal resilience by epigenetically regulating neuroprotective pathways. In rat hippocampal neurons, lithium increased viability against glutamate-induced cytotoxicity while upregulating BDNF, Bcl2, and Bcl-XL expression, and suppressing Bax, Bad, and caspase-3. Importantly, lithium selectively increased transcription of BDNF exon IV by hypomethylating its promoter region, suggesting that epigenetic modulation may further contribute to lithium-mediated protection against stress-related neuronal damage and senescence-associated processes [111].

Notably, the direct effects of lithium on senescence may be strongly cell-type-dependent. In endothelial cells, lithium down-regulates p53 and induces a senescent-like phenotype, characterized by a marked increase in MMP-1 expression, a senescence-associated marker of extracellular matrix remodeling. Interestingly, this effect was not associated with canonical GSK-3β inhibition, further supporting the notion that lithium may regulate senescence through alternative signaling pathways. Moreover, MMP-1 upregulation was shown to actively reinforce endothelial senescence, as exogenous MMP-1 enhanced the number of SA-β-Gal-positive cells, whereas MMP-1 silencing attenuated lithium-induced senescence [112].

In contrast to these findings, other studies suggest that lithium may exert context-dependent anti-senescent and regenerative effects, particularly when combined with epigenetic and pro-regenerative compounds. A recent study investigating pharmacological partial reprogramming in replicatively senescent endothelial cells demonstrated that a cocktail comprising lithium carbonate, valproic acid, and tranilast improved multiple hallmarks of endothelial dysfunction and senescence. Treatment with low-dose lithium carbonate (0.3 mM) in combination with valproic acid (0.5 mM) and tranilast (30 μM) reduced the proportion of SA-β-Gal-positive cells, enhanced proliferation, and restored migratory and angiogenic capacity to levels observed in non-senescent endothelial cells. Moreover, treated cells exhibited reduced expression of key senescence-associated regulators, including p14, p16, and p53; fewer DNA double-strand breaks; and attenuated SASP, as reflected by lower TNF-α, IL-6, and IL-8 expression. Importantly, these functional improvements occurred without loss of endothelial identity or adverse effects on non-senescent endothelial cells. Mechanistically, the beneficial effects were attributed to a transient induction of Yamanaka factor-mediated partial reprogramming, suggesting that lithium may support cellular rejuvenation under specific pharmacological and temporal conditions [113]. Notably, lithium was not administered as a monotherapy in this study, making it difficult to distinguish its direct contribution from synergistic interactions with valproic acid and tranilast. Representative experimental studies examining lithium-related modulation of cellular senescence and senescence-associated pathways are summarized in Table 1.

### 3.2. Oxidative Stress, Mitochondrial Homeostasis, DNA Repair, and Telomere Maintenance

Further support for the antioxidant-mediated anti-senescent effects of lithium comes from studies conducted in healthy human volunteers, in which lithium administration modulated systemic oxidative stress parameters even in the absence of psychiatric or neurodegenerative pathology. Oxidative stress is considered a major driver of cellular senescence, particularly in neuronal tissues, where excessive ROS promote lipid peroxidation, DNA damage, mitochondrial dysfunction, and activation of pro-senescent signaling pathways [114]. In healthy subjects receiving therapeutic lithium doses for approximately 2–4 weeks, lithium significantly reduced superoxide dismutase (SOD) activity and decreased the SOD/catalase (CAT) ratio, while CAT activity itself remained unchanged. The reduction in the SOD/CAT ratio was interpreted as reflecting lower intracellular hydrogen peroxide accumulation and, consequently, reduced oxidative stress burden. Importantly, these biochemical alterations occurred without significant changes in thiobarbituric acid-reactive substances (TBARSs), suggesting that lithium primarily modulates upstream ROS-processing pathways before overt lipid peroxidation becomes detectable [115]. In an animal model of amphetamine-induced mania, lithium was shown to prevent and partially reverse lisdexamfetamine- and d-amphetamine-associated hyperlocomotion together with oxidative stress-related alterations in the rat brain, including glutathione depletion and increased lipid peroxidation, particularly within the prefrontal cortex and striatum [116].

Additional studies employing lithium in combination with other compounds further indicate that lithium-containing treatment regimens may modulate mechanisms closely associated with cellular aging and senescence, particularly oxidative stress, excitotoxicity, and macromolecular damage. In primary cultured rat cerebral cortical cells, chronic treatment with lithium and valproate at therapeutically relevant concentrations significantly attenuated glutamate-induced intracellular calcium accumulation, lipid peroxidation, protein oxidation, DNA fragmentation, and cell death. Importantly, the treatment did not alter basal oxidative stress parameters, suggesting that the protective effects emerged predominantly under pathological cellular stress conditions [117].

Mitochondrial homeostasis may represent another important component of lithium-mediated protection against senescence-associated cellular dysfunction. Lithium has been reported to influence mitochondrial function and biogenesis, intracellular and mitochondrial Ca^2+^ homeostasis, oxidative metabolism, and autophagy-related quality-control mechanisms [103]. Through these interconnected effects, lithium may improve cellular bioenergetics, limit excessive ROS generation, and enhance resilience to chronic metabolic and oxidative stress. Such actions are particularly relevant to the CNS, where mitochondrial dysfunction and impaired mitophagy can reinforce DNA damage, inflammatory signaling, and senescence-like phenotypes. However, direct evidence demonstrating that lithium-mediated improvement of mitochondrial function is sufficient to suppress established cellular senescence remains limited; therefore, mitochondrial effects should currently be regarded as supportive mechanistic evidence rather than proof of senostatic activity.

Oxidative stress is connected with telomere shortening [44], a potential marker of accelerated biological aging in BD [45]. Chronic lithium administration has emerged as a potential modulator of this aging-related biomarker. Several studies have demonstrated that lithium treatment is associated with longer telomeres in BD patients [118,119], suggesting a protective effect against telomere erosion and cellular senescence. It was also found that the duration of lithium administration correlates with telomerase reverse transcriptase (TERT) expression, the enzyme that contributes to telomere elongation [120].

Furthermore, lithium use was also associated with reduced brain-PAD values [52], indicating a broader potential effect on biological brain aging. Given the well-established relationship between oxidative stress, genomic instability, and telomere shortening, these findings support the hypothesis that lithium may exert anti-senescent effects, at least in part, by attenuating oxidative stress and preserving telomere integrity [84].

Representative translational and clinical studies linking lithium treatment with biological aging and senescence-related outcomes are summarized in Table 2.

### 3.3. Effects on Senescence-Associated Secretory Phenotype (SASP)

Chronic stress dysregulation increases pro-inflammatory cytokines (IL-1β, IL-6, TNF-α), oxidative stress, and neurobiological damage [121], contributing to neuronal atrophy, synaptic dysfunction, and cell death, which lithium counteracts [122]. SASP links inflammation to cellular aging, and lithium may reduce pro-inflammatory signaling, thereby supporting immunomodulatory and senostatic effects [121].

Clinical and experimental data show that BD is characterized by elevated levels of TNF-α, IL-1β, and IL-6 during mood episodes, whereas lithium reduces these cytokines and promotes anti-inflammatory mediators such as IL-10 [123,124]. These effects result from lithium’s modulation of intracellular signaling pathways regulating immune responses [123]. In line with the role of inflammation in biological aging, recent findings indicate that older adults with BD exhibit a greater burden of cellular senescence, as reflected by higher SASP index scores than non-psychiatric controls. The SASP index, based on circulating proteins associated with senescence, was significantly elevated in BD, independent of age, sex, psychopathology, and physical health status. Moreover, higher SASP scores were associated with older age, greater depressive symptom severity, and increased somatic comorbidity, suggesting a link between senescence-related processes and clinical as well as systemic disease burden in BD [32]. GSK-3 inhibition may represent one contributor to lithium’s anti-inflammatory and SASP-modulating effects; however, the available evidence supports a multifactorial mechanism and does not establish GSK-3 inhibition as a specific or sufficient senostatic pathway [124]. The drug also activates the Wnt/β-catenin pathway and reduces microglial activation and neuro-inflammation [123,125,126].

In vitro, lithium reduces pro-inflammatory cytokine secretion in lipopolysaccharide (LPS)-stimulated cells, promoting an anti-inflammatory phenotype. It lowers IL-2, likely by inhibiting pro-caspase-1, and reduces IL-1β without affecting IL-6 [127,128]. Lithium also modulates the function and survival of T lymphocytes derived from patients with bipolar disorder [123].

In an AD mouse model, chronic lithium administration significantly reduced elevated levels of TNF-α, TNF receptor 1, and MARCO in the brain, indicating broad suppression of pro-inflammatory signaling [123]. Similarly, lithium attenuates astrocyte-mediated inflammation by inhibiting TLR4 expression and reducing LPS-induced IL-6 production, highlighting its role in modulating innate immune signaling pathways in the CNS [129]. Moreover, lithium reduces arachidonic acid production, a key component of the inflammatory cascade, and limits microglial activation following injury or stress [125].

Further evidence from autoimmune and inflammatory models supports the involvement of GSK-3-dependent signaling as one of several mechanisms contributing to lithium’s immunomodulatory effects. Lithium treatment suppresses pro-inflammatory cytokine production in experimental autoimmune encephalomyelitis (EAE), and reduces leukocyte infiltration and demyelination [130]. Some studies report increased IFN-γ levels and decreased IL-4 levels in lithium-treated healthy models, suggesting a potential pro-inflammatory shift under non-pathological conditions [127]. Additionally, elevated TNF-α levels have been observed in certain cohorts of lithium-treated bipolar patients, indicating variability in clinical response [124].

### 3.4. Modulation of Microglial Activation and Phenotype

In response to inflammation, microglia become activated, producing proinflammatory cytokines, nitric oxide (NO), and prostaglandins that exacerbate neuronal damage [101,131]. They polarize into proinflammatory M1 or neuroprotective M2 phenotypes; chronic stimuli (e.g., Aβ, phosphorylated tau) favor M1, sustaining inflammation and neurodegeneration [101]. In vitro and in vivo studies demonstrate that lithium attenuates microglial activation and reduces the production of proinflammatory cytokines, especially under LPS-induced inflammatory conditions [129,132]. Lithium suppresses innate immune response by downregulating Toll-like receptor 4 (TLR4) via the phosphoinositide 3-kinase/protein kinase B (PI3K/Akt) pathway, leading to FoxO1 inactivation and reduced inflammatory signaling [133]. Beyond suppressing inflammatory mediators, lithium modulates glial dynamics by reducing astrocyte proliferation while enhancing microglial survival and proliferation in vitro [131]. Moreover, lithium attenuates broader inflammatory cascades, including arachidonic acid metabolism, and reduces cytokine production in response to inflammatory stimuli [132]. In an animal model of AD, lithium treatment decreases neuroinflammatory markers, including IL-6, CXCL1, and microglial surface receptors (e.g., Trem2), and reduces microglial recruitment to affected neuronal regions [101].

In Parkinson’s disease, activated microglia and elevated proinflammatory cytokine levels are consistently observed in the substantia nigra, contributing to dopaminergic neuronal loss. Key signaling molecules such as GSK-3β further amplify this process by promoting NF-κB-mediated transcription of inflammatory mediators, thereby sustaining a detrimental feedback loop between neuronal injury and microglial activation [134,135]. Lithium’s inhibitory effects on GSK-3β may contribute to the disruption of this inflammatory cycle; however, its neuroinflammatory actions are likely mediated through multiple parallel signaling pathways. Moreover, lithium’s immunomodulatory effects are evident in demyelinating conditions such as EAE, where treatment reduces leukocyte infiltration, demyelination, and microglial activation, with potential implications for remyelination processes [136]. Although lithium also influences purinergic signaling, its effects on microglial inflammatory responses in this context appear more limited, indicating cell-type-specific mechanisms of action [85].

### 3.5. Effects on Innate Immune Signaling

Lithium exerts context-dependent effects, showing both pro- and anti-inflammatory properties. In epithelial cells, lithium rapidly activates NF-κB, leading to increased DNA binding and transcriptional activity, as well as activation of MAPK pathways (p38, ERK), even in the absence of classical inflammatory stimuli [137]. NF-κB activation downstream of pattern recognition receptors such as TLR4, TLR7, and RIG-I drives cytokine and chemokine production, positioning this pathway as a central mediator of innate immune response. Conversely, lithium can also inhibit NF-κB signaling by upregulating IκBα, preventing NF-κB nuclear translocation, and reducing inflammatory gene expression [138]. It further suppresses TLR4-mediated signaling by promoting p105 degradation, activating ERK, and reducing NF-κB activity, thereby decreasing pro-inflammatory responses and increasing macrophage apoptosis [139]. Crosstalk among TLRs, JAK/STAT, and other signaling pathways further shapes inflammatory outcomes, and dysregulation of this network contributes to chronic inflammation [140]. In the CNS, lithium influences TLR-related neuroinflammation and modulates immune cell activity and cytokine production, although clinical effects remain variable [141,142].

## 4. Conclusions and Future Perspectives

**Figure 2:**
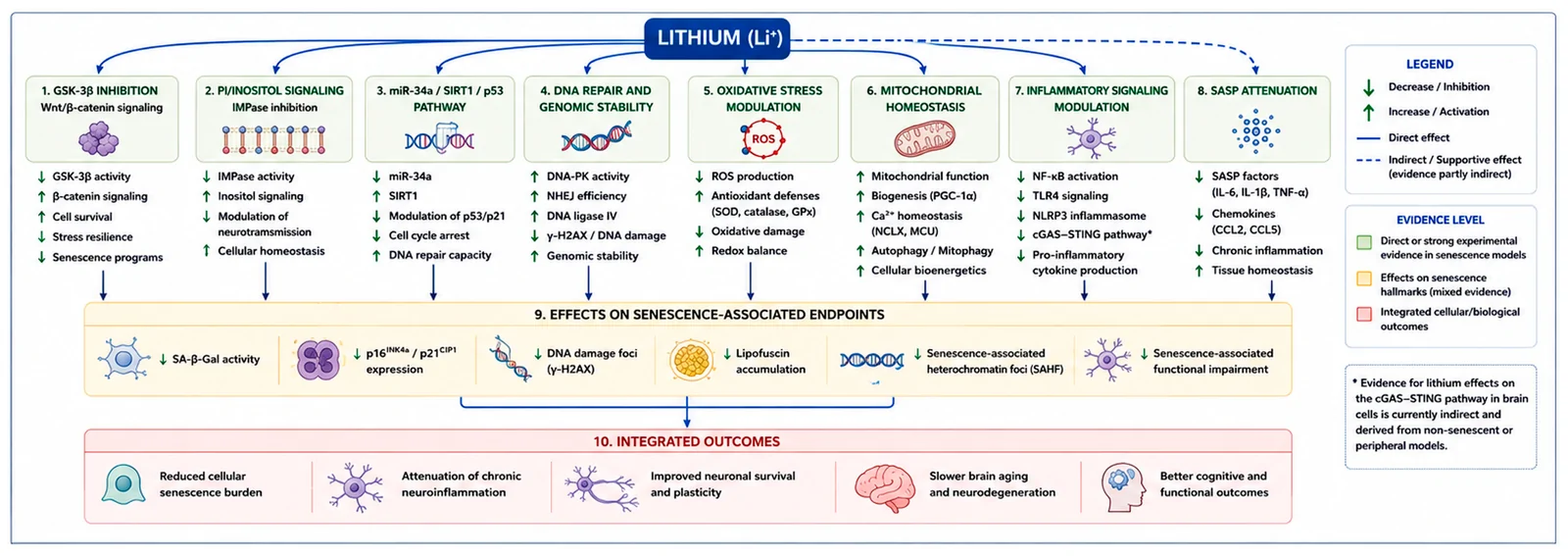
Proposed senostatic mechanisms of lithium in the aging brain and bipolar disorder. The figure summarizes the main molecular pathways potentially contributing to the senostatic effects of lithium, including GSK-3β inhibition, phosphatidylinositol signaling, miR-34a/SIRT1/p53 regulation, enhancement of DNA repair and genomic stability, reduction of oxidative stress, improvement of mitochondrial homeostasis, modulation of inflammatory signaling, and attenuation of the senescence-associated secretory phenotype (SASP). These mechanisms converge on senescence-associated endpoints, including SA-β-Gal activity, p16/p21 expression, persistent DNA damage, lipofuscin accumulation, senescence-associated heterochromatin changes, and functional impairment. The figure distinguishes more direct evidence from supportive or mechanistically indirect evidence; in particular, the involvement of certain inflammatory pathways, including cGAS–STING, should be interpreted with caution because direct evidence in lithium-treated senescent brain cells remains limited.

The question of whether lithium may be considered a senostatic agent opens an important and conceptually novel perspective on its role in psychiatry and neurobiology (Figure 2). The available evidence suggests that lithium influences multiple processes closely associated with cellular senescence, including oxidative stress, chronic inflammation, mitochondrial dysfunction, genomic instability, and impaired cellular resilience. Importantly, many of these effects appear to converge not on elimination of senescent cells, but rather on attenuation of senescence-associated dysfunction and inflammatory signaling. In this regard, lithium fits more closely within the conceptual framework of senostatic rather than senolytic interventions.

At the same time, the current evidence remains insufficient to definitively classify lithium as a true senostatic compound. Existing studies are highly heterogeneous in their experimental models, lithium concentrations, exposure durations, and senescence markers used for evaluation. Moreover, senescence itself is increasingly recognized as a dynamic, context-dependent biological state rather than a single, definable phenotype. This complexity is particularly relevant in the CNS, where post-mitotic neurons frequently exhibit senescence-like adaptations that differ substantially from canonical senescence observed in proliferative cells. Consequently, interpretation of lithium’s effects on “senescence” requires considerable caution.

Importantly, lithium does not uniformly suppress senescence-associated phenotypes across all experimental settings. Some studies indicate potentially pro-senescent or maladaptive effects, particularly in endothelial cells, while others demonstrate protective, regenerative, or anti-inflammatory actions. These discrepancies likely reflect cell-type specificity and the dual nature of stress-response pathways, in which partial activation may be adaptive under some conditions but detrimental under others. It also remains unclear to what extent lithium acts directly on senescence-regulating mechanisms versus indirectly through broader stabilization of cellular homeostasis.

Nevertheless, lithium remains uniquely positioned in senotherapeutic research due to its established clinical use, long-term safety data, and pleiotropic biological actions. Future studies should move beyond isolated biomarkers and focus on integrated models of brain aging that combine molecular, cellular, neuroimaging, and clinical dimensions. Determining whether lithium truly modifies senescence-driven neuroprogression—or merely mitigates some of its downstream consequences—will be essential for defining its place within emerging senotherapeutic strategies for neuropsychiatric disorders.

## Figures and Tables

**Table 1:** Representative experimental studies on the effects of lithium on cellular senescence and senescence-related pathways. This table summarizes selected experimental studies investigating the effects of lithium in cellular and preclinical models relevant to senescence. The included studies illustrate direct effects on established senescence-associated markers, as well as supportive effects on pathways closely linked to the senescent phenotype, including oxidative stress, DNA damage and repair, mitochondrial function, inflammatory signaling, and cellular resilience. The table also highlights differences in experimental models, lithium exposure, and the context-dependent nature of the observed effects.

Study	Model and Lithium Exposure	Senescence-Related Endpoints	Main Finding and Mechanistic Interpretation	Key Limitation
Viel et al., 2020 [105]	Human iPSC-derived astrocytes; amyloid-beta-induced senescence 2.5 µM lithium	SA-beta-Gal; p16; p21; Ser9-GSK-3beta phosphorylation	Lithium reduced SA-beta-Gal, p16, and p21 without a significant change in Ser9-GSK-3beta phosphorylation. Direct anti-senescence signal; suggests a GSK-3beta-independent mechanism at microdose exposure.	Single in vitro astrocyte model; low-dose conditions may not generalize to therapeutic concentrations.
Zmijewski & Jope, 2004 [106]	WI-38 human fibroblasts; replicative senescence 20 mM LiCl	Nuclear GSK-3; p53; p21	High LiCl reduced GSK-3-dependent nuclear accumulation of p53 and p21. Supports a GSK-3-dependent effect on senescence-associated cell-cycle signaling.	Very high lithium concentration and a non-neural cell model.
Tufekci et al., 2021 [107]	Neuronal model of H2O2-induced stress-induced premature senescence Lithium treatment; concentration as reported in study	SA-beta-Gal; lipofuscin; SAHF; p53; p21; p16; SIRT1	Lithium attenuated multiple senescence hallmarks and modulated the miR-34a/SIRT1/p53 axis. Provides direct evidence for multi-marker anti-senescent activity involving epigenetic/post-transcriptional regulation.	In vitro neuronal stress model; clinical relevance remains indirect.
Yang et al., 2016 [109]	Ischemic retinal neurocytes Lithium pretreatment	gamma-H2AX; DNA ligase IV	Lithium reduced DNA double-strand-break markers and increased ligase IV expression. Supports enhanced genomic stability through NHEJ-related DNA repair.	Measures genomic protection rather than cellular senescence directly.
Yang et al., 2009 [110]	Mouse hippocampal neurons after irradiation Lithium treatment	gamma-H2AX; DNA-PK Thr2609 foci; DNA end-joining; apoptosis	Lithium enhanced DNA-PK-dependent NHEJ and accelerated repair of double-strand breaks. Mechanistic support for lithium-mediated genomic protection relevant to senescence prevention.	Indirect senescence evidence; injury/irradiation model.
Struewing et al., 2009 [112]	Endothelial cells Lithium treatment	MMP-1; SA-beta-Gal; p53	Lithium induced a senescent-like phenotype associated with MMP-1 upregulation despite reduced p53. Demonstrates cell-type-dependent and potentially pro-senescent effects of lithium.	Non-CNS endothelial model; highlights context dependence.
Kalies et al., 2025 [113]	Replicatively senescent endothelial cells; pharmacological partial reprogramming 0.3 mM lithium carbonate + valproic acid 0.5 mM + tranilast 30 µM	SA-beta-Gal; p14; p16; p53; DNA breaks; TNF-alpha; IL-6; IL-8; proliferation/migration	Combination treatment reduced senescence markers and SASP and restored endothelial function. Supports senescence reversal/regeneration in a multi-drug context.	Lithium was not used as monotherapy; its individual contribution cannot be isolated.

**Table 2:** Representative translational and clinical studies linking lithium treatment with biological aging and senescence-related outcomes. This table summarizes selected translational and clinical studies evaluating associations between lithium exposure and markers of biological aging or senescence-related processes. The outcomes include telomere length, telomerase reverse transcriptase expression, SASP-related biomarkers, brain-predicted age difference, cognitive measures, and dementia-related outcomes. These findings should be interpreted as translational evidence of biological aging and senescence-associated processes rather than as direct proof of cellular senostatic activity.

Study	Population and Lithium Exposure	Outcome	Main Finding and Relevance to Aging	Interpretation/Limitation
Martinsson et al., 2013 [118]	Patients with bipolar disorder Long-term lithium treatment	Leukocyte telomere length	Long-term lithium treatment was associated with longer leukocyte telomeres. Telomere preservation is consistent with slower cellular aging.	Translational aging marker; not direct evidence of cellular senostasis.
Squassina et al., 2016 [119]	Patients with bipolar disorder Duration of lithium treatment	Leukocyte telomere length	Telomere length positively correlated with duration of lithium treatment. Supports a duration-related association between lithium exposure and telomere maintenance.	Observational association; residual confounding and reverse causation cannot be excluded.
Lundberg et al., 2020 [120]	Patients with bipolar disorder Duration of lithium treatment	TERT expression	TERT expression positively correlated with duration of lithium treatment. Suggests modulation of telomere-maintenance pathways.	Peripheral biomarker; does not demonstrate senostasis in CNS cells.
Tessema et al., 2024 [32]	Older adults with bipolar disorder Clinical status; lithium not the sole exposure variable	Circulating SASP index	Older adults with BD showed higher SASP scores; higher scores were associated with age, depressive symptoms and somatic burden. Defines a clinically relevant senescence-associated inflammatory phenotype in BD.	Supports disease context rather than a specific lithium treatment effect.
Topolski et al., 2026 [52]	Patients with bipolar disorder Lithium exposure in clinical cohort	Brain-predicted age difference (brain-PAD)	Lithium use was associated with reduced brain-PAD. Suggests attenuation of accelerated biological brain aging.	Neuroimaging surrogate; observational and not a direct cellular senescence measure.
Forlenza et al., 2011 [90]	Amnestic mild cognitive impairment Long-term lithium treatment	Cognition/function; phosphorylated tau	Lithium was associated with reduced phosphorylated tau and stabilization of cognitive and functional performance. Links lithium neuroprotection with clinically relevant aging outcomes.	Small clinical trial; outcome is neuroprotection rather than direct senostasis.
Kessing et al., 2010 [89]	Patients with bipolar disorder Long-term lithium treatment	Dementia risk	Long-term lithium treatment was associated with a lower rate of dementia than treatment with several other psychotropic classes. Supports a possible long-term neuroprotective effect relevant to brain aging.	Observational data; cannot establish a senostatic mechanism.

## Data Availability

No new data were created or analyzed in this study. Data sharing is not applicable to this article.

## Abbreviations

AD—Alzheimer’s disease; aMCI—amnestic mild cognitive impairment; ATM/ATR—ataxia-telangiectasia mutated/ATM and Rad3-related; BBB—blood–brain barrier; Bcl-XL—B-cell lymphoma-extra large; BD—bipolar disorder; BDNF—brain-derived neurotrophic factor; brain-PAD—brain-predicted age difference; CAT—catalase; CCL—C-C motif chemokine ligand; CCR5—C-C chemokine receptor type 5; CDK4/6—cyclin-dependent kinase 4/6; cGAS-STING—cyclic GMP-AMP synthase–stimulator of interferon genes; CNS—central nervous system; CREB1—cAMP response element-binding protein 1; CXCL1—C-X-C motif chemokine ligand 1; DAMP—damage-associated molecular pattern; DDR—DNA damage response; DNA-PK—DNA-dependent protein kinase; DPP4—dipeptidyl peptidase 4; EAE—experimental autoimmune encephalomyelitis; ER—excellent lithium responder; ERK—extracellular signal-regulated kinase; GSK-3β—glycogen synthase kinase-3 beta; HMGB1—high-mobility group box 1; IFN-γ—interferon gamma; IL—interleukin; iPSC—induced pluripotent stem cell; JAK/STAT—Janus kinase/signal transducer and activator of transcription; LPS—lipopolysaccharide; MAPK—mitogen-activated protein kinase; MARCO—macrophage receptor with collagenous structure; MCI—mild cognitive impairment; miR-34a—microRNA-34a; MMP-1—matrix metalloproteinase-1; mTOR—mechanistic target of rapamycin; NAD^+^—nicotinamide adenine dinucleotide; NF-κB—nuclear factor kappa B; NHEJ—non-homologous end joining; NLRP3—NOD-like receptor family pyrin domain-containing 3; NO—nitric oxide; OPC—oligodendrocyte progenitor cell; p53—tumor protein p53; PD—Parkinson’s disease; PI—phosphatidylinositol; PI3K/Akt—phosphoinositide 3-kinase/protein kinase B; Rb—retinoblastoma protein; RIG-I—retinoic acid-inducible gene I; ROS—reactive oxygen species; RR—risk ratio; SA-β-Gal—senescence-associated beta-galactosidase; SAHF—senescence-associated heterochromatin foci; SASP—senescence-associated secretory phenotype; SIPS—stress-induced premature senescence; SIRT1—sirtuin 1; SOD—superoxide dismutase; TBARS—thiobarbituric acid-reactive substances; TERT—telomerase reverse transcriptase; TLR—Toll-like receptor; TNF-α—tumor necrosis factor alpha; TREM2—triggering receptor expressed on myeloid cells 2; uPAR—urokinase plasminogen activator receptor; VSMC—vascular smooth muscle cell.

## References

[1] Huang W., Hickson L.J., Eirin A., Kirkland J.L., Lerman L.O. (2022). Cellular senescence: The good, the bad and the unknown. Nat. Rev. Nephrol..

[2] Ajoolabady A., Pratico D., Bahijri S., Tuomilehto J., Uversky V.N., Ren J. (2025). Hallmarks of cellular senescence: Biology, mechanisms, regulations. Exp. Mol. Med..

[3] Lucas V., Cavadas C., Aveleira C.A. (2023). Cellular Senescence: From Mechanisms to Current Biomarkers and Senotherapies. Pharmacol. Rev..

[4] Rossi M., Abdelmohsen K. (2021). The Emergence of Senescent Surface Biomarkers as Senotherapeutic Targets. Cells.

[5] Alqahtani S., Alqahtani T., Venkatesan K., Sivadasan D., Ahmed R., Sirag N., Elfadil H., Abdullah Mohamed H., T.A. H., Elsayed Ahmed R. (2025). SASP Modulation for Cellular Rejuvenation and Tissue Homeostasis: Therapeutic Strategies and Molecular Insights. Cells.

[6] Kaur J., Farr J.N. (2020). Cellular senescence in age-related disorders. Transl. Res..

[7] Mydlak C., Książek K. (2025). Therapeutic implications of senotherapy in neurodegenerative diseases: From molecular basis to clinical perspectives. Pharmacother. Psychiatry Neurol..

[8] Feng H., Li J., Wang H., Wei Z., Feng S. (2024). Senescence- and Immunity-Related Changes in the Central Nervous System: A Comprehensive Review. Aging Dis..

[9] Sikora E., Bielak-Zmijewska A., Dudkowska M., Krzystyniak A., Mosieniak G., Wesierska M., Wlodarczyk J. (2021). Cellular Senescence in Brain Aging. Front. Aging Neurosci..

[10] de Luzy I.R., Lee M.K., Mobley W.C., Studer L. (2024). Lessons from inducible pluripotent stem cell models on neuronal senescence in aging and neurodegeneration. Nat. Aging.

[11] Horikawa I., Yamada L., Harris B.T., Harris C.C. (2025). Delta133p53alpha-mediated inhibition of astrocyte senescence and neurotoxicity as a possible therapeutic approach for neurodegenerative diseases. Neuroscience.

[12] Alshaebi F., Sciortino A., Kayed R. (2025). The Role of Glial Cell Senescence in Alzheimer’s Disease. J. Neurochem..

[13] Gosselin D., Rivest S. (2018). Getting Too Old Too Quickly for Their Job: Senescent Glial Cells Promote Neurodegeneration. Neuron.

[14] Zhong S., Zhou Q., Yang J., Zhang Z., Zhang X., Liu J., Chang X., Wang H. (2024). Relationship between the cGAS−STING and NF-κB pathways-role in neurotoxicity. Biomed. Pharmacother..

[15] Chen H., Sun Y.Y., Li Q.F., Du Y.T., Hu N.N., Sui A.R., Luo X.Q., Huang X., Zhu C., Yang G. (2025). Impaired macroautophagy in oligodendrocyte precursor cells suppresses neuronal plasticity via a senescence-associated signaling. Sci. Adv..

[16] Lennon M.J., Karvelas N., Ganesh A., Whitehead S., Sorond F.A., Duran Laforet V., Head E., Arfanakis K., Kolachalama V.B., Liu X. (2025). The future of biomarkers for vascular contributions to cognitive impairment and dementia (VCID): Proceedings of the 2025 annual workshop of the Albert research institute for white matter and cognition. Geroscience.

[17] Atkinson J., Dokiburra A., Groover H., Godbout J.P., Segal B.M., Zhang Y. (2025). Targeting senescent microglia in progressive multiple sclerosis: A geroscience-informed approach. Front. Immunol..

[18] Gross P.S., Duran-Laforet V., Ho L.T., Melchor G.S., Zia S., Manavi Z., Barclay W.E., Lee S.H., Shults N., Selva S. (2025). Senescent-like microglia limit remyelination through the senescence associated secretory phenotype. Nat. Commun..

[19] Bussian T.J., Aziz A., Meyer C.F., Swenson B.L., van Deursen J.M., Baker D.J. (2018). Clearance of senescent glial cells prevents tau-dependent pathology and cognitive decline. Nature.

[20] Graves S.I., Meyer C.F., Jeganathan K.B., Baker D.J. (2025). p16-expressing microglia and endothelial cells promote tauopathy and neurovascular abnormalities in PS19 mice. Neuron.

[21] Matsudaira T., Nakano S., Konishi Y., Kawamoto S., Uemura K., Kondo T., Sakurai K., Ozawa T., Hikida T., Komine O. (2023). Cellular senescence in white matter microglia is induced during ageing in mice and exacerbates the neuroinflammatory phenotype. Commun. Biol..

[22] Mani S., Wasnik S., Shandilya C., Srivastava V., Khan S., Singh K.K. (2025). Pathogenic synergy: Dysfunctional mitochondria and neuroinflammation in neurodegenerative diseases associated with aging. Front. Aging.

[23] Tamatta R., Pai V., Jaiswal C., Singh I., Singh A.K. (2025). Neuroinflammaging and the Immune Landscape: The Role of Autophagy and Senescence in Aging Brain. Biogerontology.

[24] Wang Y., Kuca K., You L., Nepovimova E., Heger Z., Valko M., Adam V., Wu Q., Jomova K. (2024). The role of cellular senescence in neurodegenerative diseases. Arch. Toxicol..

[25] Glück S., Guey B., Gulen M.F., Wolter K., Kang T.-W., Schmacke N.A., Bridgeman A., Rehwinkel J., Zender L., Ablasser A. (2017). Innate immune sensing of cytosolic chromatin fragments through cGAS promotes senescence. Nat. Cell Biol..

[26] Dou Z., Ghosh K., Vizioli M.G., Zhu J., Sen P., Wangensteen K.J., Simithy J., Lan Y., Lin Y., Zhou Z. (2017). Cytoplasmic chromatin triggers inflammation in senescence and cancer. Nature.

[27] Tan M.-S., Yu J.-T., Jiang T., Zhu X.-C., Tan L. (2013). The NLRP3 Inflammasome in Alzheimer’s Disease. Mol. Neurobiol..

[28] Ulland T.K., Colonna M. (2018). TREM2—A key player in microglial biology and Alzheimer disease. Nat. Rev. Neurol..

[29] Colon Z.A., Chan S.C., Maguire-Zeiss K.A. (2025). Age-related inflammatory changes and perineuronal net dynamics: Implications for aging. J. Neuroinflamm..

[30] Yousef H., Czupalla C.J., Lee D., Chen M.B., Burke A.N., Zera K.A., Zandstra J., Berber E., Lehallier B., Mathur V. (2019). Aged blood impairs hippocampal neural precursor activity and activates microglia via brain endothelial cell VCAM1. Nat. Med..

[31] Jaganathan R., Iyaswamy A., Krishnamoorthi S., Thakur A., Durairajan S.S.K., Yang C., Wankhar D. (2025). Current aspects of targeting cellular senescence for the therapy of neurodegenerative diseases. Front. Aging Neurosci..

[32] Tessema T., Diniz B.S., Vieira E.M., Mendes-Silva A.P., Voineskos A.N., Gildengers A.G., Husain M.I., Ortiz A., Blumberger D.M., Rajji T.K. (2024). Elevated senescence-associated secretory phenotype index in late-life bipolar disorder. J. Affect. Disord..

[33] Williams D.W., Flores B.R., Xu Y., Wang Y., Yu D., Peters B.A., Adedimeji A., Wilson T.E., Merenstein D., Tien P.C. (2022). T-cell activation state differentially contributes to neuropsychiatric complications in women with HIV. Brain Behav. Immun. Health.

[34] Diniz B.S., Reynolds C.F., Sibille E., Lin C.W., Tseng G., Lotrich F., Aizenstein H.J., Butters M.A. (2017). Enhanced Molecular Aging in Late-Life Depression: The Senescent-Associated Secretory Phenotype. Am. J. Geriatr. Psychiatry.

[35] Diniz B.S., Vieira E.M., Mendes-Silva A.P., Bowie C.R., Butters M.A., Fischer C.E., Flint A., Herrmann N., Kennedy J., Lanctot K.L. (2021). Mild cognitive impairment and major depressive disorder are associated with molecular senescence abnormalities in older adults. Alzheimers Dement..

[36] Diniz B.S., Mulsant B.H., Reynolds C.F., Blumberger D.M., Karp J.F., Butters M.A., Mendes-Silva A.P., Vieira E.L., Tseng G., Lenze E.J. (2022). Association of Molecular Senescence Markers in Late-Life Depression with Clinical Characteristics and Treatment Outcome. JAMA Netw. Open.

[37] Seitz-Holland J., Mulsant B.H., Reynolds C.F., Blumberger D.M., Karp J.F., Butters M.A., Mendes-Silva A.P., Vieira E.L., Tseng G., Lenze E.J. (2023). Major depression, physical health and molecular senescence markers abnormalities. Nat. Ment. Health.

[38] Yirmiya R., Goshen I. (2011). Immune modulation of learning, memory, neural plasticity and neurogenesis. Brain Behav. Immun..

[39] Miller A.H., Raison C.L. (2016). The role of inflammation in depression: From evolutionary imperative to modern treatment target. Nat. Rev. Immunol..

[40] Koo J.W., Duman R.S. (2008). IL-1β is an essential mediator of the antineurogenic and anhedonic effects of stress. Proc. Natl. Acad. Sci. USA.

[41] Franceschi C., Campisi J. (2014). Chronic Inflammation (Inflammaging) and Its Potential Contribution to Age-Associated Diseases. J. Gerontol. Ser. A Biol. Sci. Med. Sci..

[42] Walker E.R., McGee R.E., Druss B.G. (2015). Mortality in mental disorders and global disease burden implications: A systematic review and meta-analysis. JAMA Psychiatry.

[43] Jiang C., Ge X., Wu C., Zhang X., Fang X. (2026). Accelerated aging in psychiatric disorders: Evidence from epigenetic clocks. Prog. Neuropsychopharmacol. Biol. Psychiatry.

[44] von Zglinicki T. (2002). Oxidative stress shortens telomeres. Trends Biochem. Sci..

[45] Liguori I., Russo G., Curcio F., Bulli G., Aran L., Della-Morte D., Gargiulo G., Testa G., Cacciatore F., Bonaduce D. (2018). Oxidative stress, aging, and diseases. Clin. Interv. Aging.

[46] Vasconcelos-Moreno M.P., Fries G.R., Gubert C., Dos Santos B., Fijtman A., Sartori J., Ferrari P., Grun L.K., Parisi M.M., Guma F. (2017). Telomere Length, Oxidative Stress, Inflammation and BDNF Levels in Siblings of Patients with Bipolar Disorder: Implications for Accelerated Cellular Aging. Int. J. Neuropsychopharmacol..

[47] Barbe-Tuana F.M., Parisi M.M., Panizzutti B.S., Fries G.R., Grun L.K., Guma F.T., Kapczinski F., Berk M., Gama C.S., Rosa A.R. (2016). Shortened telomere length in bipolar disorder: A comparison of the early and late stages of disease. Braz. J. Psychiatry.

[48] Ferensztajn-Rochowiak E., Kurczewska E., Rubis B., Lulkiewicz M., Holysz H., Rybakowski F., Rybakowski J.K. (2021). Decreased leucocyte telomere length in male patients with chronic bipolar disorder: Lack of effect of long-term lithium treatment. Acta Neuropsychiatr..

[49] Huang Y.C., Wang L.J., Tseng P.T., Hung C.F., Lin P.Y. (2018). Leukocyte telomere length in patients with bipolar disorder: An updated meta-analysis and subgroup analysis by mood status. Psychiatry Res..

[50] Fries G.R., Zamzow M.J., Andrews T., Pink O., Scaini G., Quevedo J. (2020). Accelerated aging in bipolar disorder: A comprehensive review of molecular findings and their clinical implications. Neurosci. Biobehav. Rev..

[51] Uzman Ozbek S., Akyol K., Bora E. (2025). Does bipolar disorder accelerate cellular aging? A systematic review and meta-analysis of telomere length. Eur. Neuropsychopharmacol..

[52] Topolski N., Barsotti E.J., Boscutti A., Harmata G.I.S., Kovacs E.H.C., Magnotta V.A., Fries G.R., Mwangi B., Gaine M.E., Wemmie J.A. (2026). Brain Age in Bipolar Disorder: Impact of Model Selection and Clinical Factors. Biol. Psychiatry Cogn. Neurosci. Neuroimaging.

[53] López-Otín C., Blasco M.A., Partridge L., Serrano M., Kroemer G. (2023). Hallmarks of aging: An expanding universe. Cell.

[54] Baker D.J., Wijshake T., Tchkonia T., LeBrasseur N.K., Childs B.G., van de Sluis B., Kirkland J.L., van Deursen J.M. (2011). Clearance of p16Ink4a-positive senescent cells delays ageing-associated disorders. Nature.

[55] Zhu M., Meng P., Ling X., Zhou L. (2020). Advancements in therapeutic drugs targeting of senescence. Ther. Adv. Chronic Dis..

[56] Boccardi V., Mecocci P. (2021). Senotherapeutics: Targeting senescent cells for the main age-related diseases. Mech. Ageing Dev..

[57] Patusco R., Gyasi K., Kaufmann A. (2026). Exploring the nexus between inflammation and mobility through the lens of healthy aging: Current scenario and future perspectives. Aging Clin. Exp. Res..

[58] Yang N.-S.-Y., Zhong W.-J., Sha H.-X., Zhang C.-Y., Jin L., Duan J.-X., Xiong J.-B., You Z.-J., Zhou Y., Guan C.-X. (2024). mtDNA-cGAS-STING axis-dependent NLRP3 inflammasome activation contributes to postoperative cognitive dysfunction induced by sevoflurane in mice. Int. J. Biol. Sci..

[59] Puglisi-Allegra S., Lazzeri G., Busceti C.L., Giorgi F.S., Biagioni F., Fornai F. (2023). Lithium engages autophagy for neuroprotection and neuroplasticity: Translational evidence for therapy. Neurosci. Biobehav. Rev..

[60] Salarda E.M., Zhao N.O., Lima C., Fries G.R. (2021). Mini-review: The anti-aging effects of lithium in bipolar disorder. Neurosci. Lett..

[61] Lange C. (1886). Om Periodiske Depressionstilstande og Deres Patogenese.

[62] Cade J.F. (1949). Lithium salts in the treatment of psychotic excitement. Med. J. Aust..

[63] Hartigan G.P. (1963). The Use of Lithium Salts in Affective Disorders. Br. J. Psychiatry.

[64] Baastrup P.C. (1964). The Use of Lithium in Manic-Depressive Psychosis. Compr. Psychiatry.

[65] Grof P., Birch N.J., Gallicchio V.S., Becker R.W. (1999). Excellent lithium responders: People whose lives have been changed by lithium prophylaxis. Lithium: 50 Years of Psychopharmacology, New Perspectives in Biomedical and Clinical Research.

[66] Rybakowski J.K., Chlopocka-Wozniak M., Suwalska A. (2001). The prophylactic effect of long-term lithium administration in bipolar patients entering treatment in the 1970s and 1980s. Bipolar Disord..

[67] Geddes J.R., Burgess S., Hawton K., Jamison K., Goodwin G.M. (2004). Long-term lithium therapy for bipolar disorder: Systematic review and meta-analysis of randomized controlled trials. Am. J. Psychiatry.

[68] Severus E., Taylor M.J., Sauer C., Pfennig A., Ritter P., Bauer M., Geddes J.R. (2014). Lithium for prevention of mood episodes in bipolar disorders: Systematic review and meta-analysis. Int. J. Bipolar Disord..

[69] Poolsup N., Li Wan Po A., de Oliveira I.R. (2000). Systematic overview of lithium treatment in acute mania. J. Clin. Pharm. Ther..

[70] De Montigny C., Grunberg F., Mayer A., Deschenes J.P. (1981). Lithium induces rapid relief of depression in tricyclic antidepressant drug non-responders. Br. J. Psychiatry.

[71] Crossley N.A., Bauer M. (2007). Acceleration and augmentation of antidepressants with lithium for depressive disorders: Two meta-analyses of randomized, placebo-controlled trials. J. Clin. Psychiatry.

[72] Cipriani A., Hawton K., Stockton S., Geddes J.R. (2013). Lithium in the prevention of suicide in mood disorders: Updated systematic review and meta-analysis. BMJ.

[73] Rybakowski J.K. (2019). Commentary: Corroboration of a Major Role for Herpes Simplex Virus Type 1 in Alzheimer’s Disease. Front. Aging Neurosci..

[74] Rybakowski J.K. (2022). Antiviral, immunomodulatory, and neuroprotective effect of lithium. J. Integr. Neurosci..

[75] Kohler-Forsberg O., Rohde C., Nierenberg A.A., Ostergaard S.D. (2022). Association of Lithium Treatment with the Risk of Osteoporosis in Patients with Bipolar Disorder. JAMA Psychiatry.

[76] Williams L.J., Agustini B., Stuart A.L., Pasco J.A., Hodge J.M., Samarasinghe R.M., Bjerkeset O., Quirk S.E., Koivumaa-Honkanen H., Honkanen R. (2024). Lithium use and bone health in women with bipolar disorder: A cross-sectional study. Acta Psychiatr. Scand..

[77] Chen P.H., Hsiao C.Y., Chiang S.J., Tsai S.Y. (2023). Association of Lithium Treatment with Reduced Left Ventricular Concentricity in Patients with Bipolar Disorder. Psychiatry Clin. Neurosci..

[78] Almeida O.P., Singulani M.P., Ford A.H., Hackett M.L., Etherton-Beer C., Flicker L., Hankey G.J., De Paula V.J.R., Penteado C.T., Forlenza O.V. (2022). Lithium and Stroke Recovery: A Systematic Review and Meta-Analysis of Stroke Models in Rodents and Human Data. Stroke.

[79] Romo-Nava F., Blom T., Cuellar-Barboza A.B., Barrera F.J., Miola A., Mori N.N., Prieto M.L., Veldic M., Singh B., Gardea-Resendez M. (2023). Clinical characterization of patients with bipolar disorder and a history of asthma: An exploratory study. J. Psychiatr. Res..

[80] Anmella G., Fico G., Lotfaliany M., Hidalgo-Mazzei D., Soto-Angona O., Gimenez-Palomo A., Amoretti S., Murru A., Radua J., Solanes A. (2021). Risk of cancer in bipolar disorder and the potential role of lithium: International collaborative systematic review and meta-analyses. Neurosci. Biobehav. Rev..

[81] Rohde C., Kohler-Forsberg O., Nierenberg A.A., Ostergaard S.D. (2023). Pharmacological treatment of bipolar disorder and risk of diabetes mellitus: A nationwide study of 30,451 patients. Bipolar Disord..

[82] Chen P.H., Tsai S.Y., Chen P.Y., Pan C.H., Su S.S., Chen C.C., Kuo C.J. (2023). Mood stabilizers and risk of all-cause, natural, and suicide mortality in bipolar disorder: A nationwide cohort study. Acta Psychiatr. Scand..

[83] Oliva V., De Prisco M., Anmella G., Valenzuela-Pascual C., Mas A., Fernandez T., Fico G., Murru A., Valenti M., Blanch J. (2025). Patterns of lithium exposure and mortality in bipolar disorder: A population-based cohort study. Eur. Psychiatry.

[84] Courtes A.C., Jha R., Topolski N., Soares J.C., Barichello T., Fries G.R. (2024). Exploring accelerated aging as a target of bipolar disorder treatment: A systematic review. J. Psychiatr. Res..

[85] Sakrajda K., Rybakowski J.K. (2025). The Mechanisms of Lithium Action: The Old and New Findings. Pharmaceuticals.

[86] Moore G.J., Bebchuk J.M., Wilds I.B., Chen G., Manji H.K. (2000). Lithium-induced increase in human brain grey matter. Lancet.

[87] Hajek T., Weiner M.W. (2016). Neuroprotective Effects of Lithium in Human Brain? Food for Thought. Curr. Alzheimer Res..

[88] Donix M., Bauer M. (2016). Population Studies of Association Between Lithium and Risk of Neurodegenerative Disorders. Curr. Alzheimer Res..

[89] Kessing L.V., Forman J.L., Andersen P.K. (2010). Does lithium protect against dementia?. Bipolar Disord..

[90] Forlenza O.V., Diniz B.S., Radanovic M., Santos F.S., Talib L.L., Gattaz W.F. (2011). Disease-modifying properties of long-term lithium treatment for amnestic mild cognitive impairment: Randomised controlled trial. Br. J. Psychiatry.

[91] Nunes M.A., Viel T.A., Buck H.S. (2013). Microdose lithium treatment stabilized cognitive impairment in patients with Alzheimer’s disease. Curr. Alzheimer Res..

[92] Forlenza O.V., Radanovic M., Talib L.L., Gattaz W.F. (2019). Clinical and biological effects of long-term lithium treatment in older adults with amnestic mild cognitive impairment: Randomised clinical trial. Br. J. Psychiatry.

[93] Damiano R.F., Loureiro J.C., Pais M.V., Pereira R.F., Corradi M.M., Di Santi T., Bezerra G.A.M., Radanovic M., Talib L.L., Forlenza O.V. (2023). Revisiting global cognitive and functional state 13 years after a clinical trial of lithium for mild cognitive impairment. Braz. J. Psychiatry.

[94] Matsunaga S., Kishi T., Annas P., Basun H., Hampel H., Iwata N. (2015). Lithium as a Treatment for Alzheimer’s Disease: A Systematic Review and Meta-Analysis. J. Alzheimers Dis..

[95] Matsunaga S., Fujishiro H., Takechi H. (2019). Efficacy and Safety of Glycogen Synthase Kinase 3 Inhibitors for Alzheimer’s Disease: A Systematic Review and Meta-Analysis. J. Alzheimers Dis..

[96] Terao I., Kodama W. (2024). Comparative efficacy, tolerability and acceptability of donanemab, lecanemab, aducanumab and lithium on cognitive function in mild cognitive impairment and Alzheimer’s disease: A systematic review and network meta-analysis. Ageing Res. Rev..

[97] Pereira da Silva A.M., de Deus O., Ribeiro F.V., Tudella G.C.N., Cabeca L.S., Silva L.L., Han M.L., Franco J.O., Costa J.G.P., Santos do Nascimento M.D.V. (2026). Efficacy and Safety of Lithium for Behavioral and Cognitive Symptoms in Alzheimer’s Disease Dementia: A Systematic Review with Frequentist and Bayesian Meta-Analysis. Am. J. Geriatr. Psychiatry.

[98] Aron L., Ngian Z.K., Qiu C., Choi J., Liang M., Drake D.M., Hamplova S.E., Lacey E.K., Roche P., Yuan M. (2025). Lithium deficiency and the onset of Alzheimer’s disease. Nature.

[99] Kessing L.V., Gerds T.A., Knudsen N.N., Jorgensen L.F., Kristiansen S.M., Voutchkova D., Ernstsen V., Schullehner J., Hansen B., Andersen P.K. (2017). Association of Lithium in Drinking Water with the Incidence of Dementia. JAMA Psychiatry.

[100] Fajardo V.A., Fajardo V.A., LeBlanc P.J., MacPherson R.E.K. (2018). Examining the Relationship between Trace Lithium in Drinking Water and the Rising Rates of Age-Adjusted Alzheimer’s Disease Mortality in Texas. J. Alzheimers Dis..

[101] Shen Y., Zhao M., Zhao P., Meng L., Zhang Y., Zhang G., Taishi Y., Sun L. (2024). Molecular mechanisms and therapeutic potential of lithium in Alzheimer’s disease: Repurposing an old class of drugs. Front. Pharmacol..

[102] Itzhaki R.F. (2018). Corroboration of a Major Role for Herpes Simplex Virus Type 1 in Alzheimer’s Disease. Front. Aging Neurosci..

[103] Godoy J.A., Mira R.G., Inestrosa N.C. (2024). Intracellular effects of lithium in aging neurons. Ageing Res. Rev..

[104] Nunes M.A., Schowe N.M., Monteiro-Silva K.C., Baraldi-Tornisielo T., Souza S.I., Balthazar J., Albuquerque M.S., Caetano A.L., Viel T.A., Buck H.S. (2015). Chronic Microdose Lithium Treatment Prevented Memory Loss and Neurohistopathological Changes in a Transgenic Mouse Model of Alzheimer’s Disease. PLoS ONE.

[105] Viel T., Chinta S., Rane A., Chamoli M., Buck H., Andersen J. (2020). Microdose lithium reduces cellular senescence in human astrocytes—A potential pharmacotherapy for COVID-19?. Aging (Albany NY).

[106] Zmijewski J.W., Jope R.S. (2004). Nuclear accumulation of glycogen synthase kinase-3 during replicative senescence of human fibroblasts. Aging Cell.

[107] Tufekci K.U., Alural B., Tarakcioglu E., San T., Genc S. (2021). Lithium inhibits oxidative stress-induced neuronal senescence through miR-34a. Mol. Biol. Rep..

[108] Zuccolo E., Badi I., Scavello F., Gambuzza I., Mancinelli L., Macri F., Tedesco C.C., Veglia F., Bonfigli A.R., Olivieri F. (2020). The microRNA-34a-Induced Senescence-Associated Secretory Phenotype (SASP) Favors Vascular Smooth Muscle Cells Calcification. Int. J. Mol. Sci..

[109] Yang Y., Wu N., Tian S., Li F., Hu H., Chen P., Cai X., Xu L., Zhang J., Chen Z. (2016). Lithium promotes DNA stability and survival of ischemic retinal neurocytes by upregulating DNA ligase IV. Cell Death Dis..

[110] Yang E.S., Wang H., Jiang G., Nowsheen S., Fu A., Hallahan D.E., Xia F. (2009). Lithium-mediated protection of hippocampal cells involves enhancement of DNA-PK-dependent repair in mice. J. Clin. Investig..

[111] Dwivedi T., Zhang H. (2014). Lithium-induced neuroprotection is associated with epigenetic modification of specific BDNF gene promoter and altered expression of apoptotic-regulatory proteins. Front. Neurosci..

[112] Struewing I.T., Durham S.N., Barnett C.D., Mao C.D. (2009). Enhanced endothelial cell senescence by lithium-induced matrix metalloproteinase-1 expression. J. Biol. Chem..

[113] Kalies K., Knopp K., Koch S., Pilowski C., Wurmbrand L., Sedding D. (2025). Restoration of angiogenic capacity in senescent endothelial cells by a pharmacological reprogramming approach. PLoS ONE.

[114] Stojanovic B., Jovanovic I., Dimitrijevic Stojanovic M., Stojanovic B.S., Kovacevic V., Radosavljevic I., Jovanovic D., Miletic Kovacevic M., Zornic N., Arsic A.A. (2025). Oxidative Stress-Driven Cellular Senescence: Mechanistic Crosstalk and Therapeutic Horizons. Antioxidants.

[115] Khairova R., Pawar R., Salvadore G., Juruena M.F., de Sousa R.T., Soeiro-de-Souza M.G., Salvador M., Zarate C.A., Gattaz W.F., Machado-Vieira R. (2012). Effects of lithium on oxidative stress parameters in healthy subjects. Mol. Med. Rep..

[116] Macedo D.S., de Lucena D.F., Queiroz A.I., Cordeiro R.C., Araujo M.M., Sousa F.C., Vasconcelos S.M., Hyphantis T.N., Quevedo J., McIntyre R.S. (2013). Effects of lithium on oxidative stress and behavioral alterations induced by lisdexamfetamine dimesylate: Relevance as an animal model of mania. Prog. Neuropsychopharmacol. Biol. Psychiatry.

[117] Shao L., Young L.T., Wang J.F. (2005). Chronic treatment with mood stabilizers lithium and valproate prevents excitotoxicity by inhibiting oxidative stress in rat cerebral cortical cells. Biol. Psychiatry.

[118] Martinsson L., Wei Y., Xu D., Melas P.A., Mathe A.A., Schalling M., Lavebratt C., Backlund L. (2013). Long-term lithium treatment in bipolar disorder is associated with longer leukocyte telomeres. Transl. Psychiatry.

[119] Squassina A., Pisanu C., Congiu D., Caria P., Frau D., Niola P., Melis C., Baggiani G., Lopez J.P., Cruceanu C. (2016). Leukocyte telomere length positively correlates with duration of lithium treatment in bipolar disorder patients. Eur. Neuropsychopharmacol..

[120] Lundberg M., Biernacka J.M., Lavebratt C., Druliner B., Ryu E., Geske J., Colby C., Boardman L., Frye M., Schalling M. (2020). Expression of telomerase reverse transcriptase positively correlates with duration of lithium treatment in bipolar disorder. Psychiatry Res..

[121] Ferensztajn-Rochowiak E., Szczepankiewicz A., Rybakowski J. (2021). Lithium treatment and stress response in bipolar disorder. Pharmacother. Psychiatry Neurol..

[122] Machado-Vieira R., Manji H.K., Zarate C.A. (2009). The role of lithium in the treatment of bipolar disorder: Convergent evidence for neurotrophic effects as a unifying hypothesis. Bipolar Disord..

[123] Habib M.Z., Ebeid M.A., El Faramawy Y., Saad S.S.T., El Magdoub H.M., Attia A.A., Aboul-Fotouh S., Abdel-Tawab A.M. (2020). Effects of lithium on cytokine neuro-inflammatory mediators, Wnt/β-catenin signaling and microglial activation in the hippocampus of chronic mild stress-exposed rats. Toxicol. Appl. Pharmacol..

[124] Chatterjee D., Beaulieu J.M. (2022). Inhibition of glycogen synthase kinase 3 by lithium, a mechanism in search of specificity. Front. Mol. Neurosci..

[125] Forlenza O.V., De-Paula V.J.R., Diniz B.S.O. (2014). Neuroprotective Effects of Lithium: Implications for the Treatment of Alzheimer’s Disease and Related Neurodegenerative Disorders. ACS Chem. Neurosci..

[126] Sakrajda K., Langwiński W., Stachowiak Z., Ziarniak K., Narożna B., Szczepankiewicz A. (2025). Immunomodulatory effect of lithium treatment on in vitro model of neuroinflammation. Neuropharmacology.

[127] Matur E., Akyol S., Toplan S., Ozdemir S., Akyazı I., Darıyerli N. (2025). Impact of Lithium on the Immune System: An Investigation of T-Cell Subpopulations and Cytokine Responses in Rats. Biol. Trace Elem. Res..

[128] Knijff E.M., Nadine Breunis M., Kupka R.W., De Wit H.J., Ruwhof C., Akkerhuis G.W., Nolen W.A., Drexhage H.A. (2007). An imbalance in the production of IL-1β and IL-6 by monocytes of bipolar patients: Restoration by lithium treatment. Bipolar Disord..

[129] Li N., Zhang X., Dong H., Zhang S., Sun J., Qian Y. (2016). Lithium Ameliorates LPS-Induced Astrocytes Activation Partly via Inhibition of Toll-Like Receptor 4 Expression. Cell Physiol. Biochem..

[130] Chiu C.-T., Chuang D.-M. (2010). Molecular actions and therapeutic potential of lithium in preclinical and clinical studies of CNS disorders. Pharmacol. Ther..

[131] Nassar A., Azab A.N. (2014). Effects of lithium on inflammation. ACS Chem. Neurosci..

[132] Diniz B.S., Machado-Vieira R., Forlenza O.V. (2013). Lithium and neuroprotection: Translational evidence and implications for the treatment of neuropsychiatric disorders. Neuropsychiatr. Dis. Treat..

[133] Dong H., Zhang X., Dai X., Lu S., Gui B., Jin W., Zhang S., Zhang S., Qian Y. (2014). Lithium ameliorates lipopolysaccharide-induced microglial activation via inhibition of toll-like receptor 4 expression by activating the PI3K/Akt/FoxO1 pathway. J. Neuroinflamm..

[134] Li D.-W., Liu Z.-Q., Wei C., Min Y., Li G.-R. (2014). Association of glycogen synthase kinase-3β with Parkinson’s disease (Review). Mol. Med. Rep..

[135] Wang H.-M., Zhang T., Li Q., Huang J.-K., Chen R.-F., Sun X.-J. (2013). Inhibition of glycogen synthase kinase-3β by lithium chloride suppresses 6-hydroxydopamine-induced inflammatory response in primary cultured astrocytes. Neurochem. Int..

[136] De Sarno P., Axtell R.C., Raman C., Roth K.A., Alessi D.R., Jope R.S. (2008). Lithium Prevents and Ameliorates Experimental Autoimmune Encephalomyelitis. J. Immunol..

[137] Németh Z.H., Deitch E.A., Szabó C., Fekete Z., Hauser C.J., Haskó G. (2002). Lithium Induces NF-κB Activation and Interleukin-8 Production in Human Intestinal Epithelial Cells. J. Biol. Chem..

[138] Makola R.T., Kgaladi J., More G.K., Jansen Van Vuren P., Paweska J.T., Matsebatlela T.M. (2021). Lithium inhibits NF-κB nuclear translocation and modulate inflammation profiles in Rift valley fever virus-infected Raw 264.7 macrophages. Virol. J..

[139] Zhang M., Jin W., Zhou X., Yu J., Lee A.J., Sun S.-C. (2009). Deregulation of Tpl2 and NF-κB signaling and induction of macrophage apoptosis by the anti-depressant drug lithium. Cell. Signal..

[140] Hu X., Chen J., Wang L., Ivashkiv L.B. (2007). Crosstalk among Jak-STAT, Toll-like receptor, and ITAM-dependent pathways in macrophage activation. J. Leukoc. Biol..

[141] Chaudhary A., Mehra P., Keshri A.K., Rawat S.S., Mishra A., Prasad A. (2024). The Emerging Role of Toll-Like Receptor-Mediated Neuroinflammatory Signals in Psychiatric Disorders and Acquired Epilepsy. Mol. Neurobiol..

[142] Szałach Ł.P., Lisowska K.A., Cubała W.J., Barbuti M., Perugi G. (2023). The immunomodulatory effect of lithium as a mechanism of action in bipolar disorder. Front. Neurosci..

